# Religiosity in patients with amyotrophic lateral sclerosis, a cross-country comparison

**DOI:** 10.1007/s11136-023-03383-4

**Published:** 2023-03-16

**Authors:** Katarzyna Ciećwierska, Dorothée Lulé, Olga Helczyk, Krzysztof Nieporęcki, Maksymilian Bielecki, Susanne Baader, Albert C. Ludolph, Magdalena Kuźma-Kozakiewicz

**Affiliations:** 1 Department of Neurology, University Clinical Centre of the Medical University of Warsaw, Warsaw, Poland; 2 Department of Neurology, University of Ulm, Ulm, Germany; 3 Department of Psychology, SWPS University of Social Sciences and Humanities, Warsaw, Poland; 4 Department of Neurology, Medical University of Warsaw, Warsaw, Poland; 5 Neurodegenerative Diseases Research Group, Medical University of Warsaw, Warsaw, Poland; 6 German Center for Neurodegenerative Diseases, Ulm Site, Ulm, Germany

**Keywords:** Amyotrophic lateral sclerosis, Religiosity, Depression, Quality of life, Pain, Culture

## Abstract

**Purpose:**

Amyotrophic lateral sclerosis (ALS) is a progressive motor impairment leading to early death. Religiousness is one of the factors potentially alleviating the psychological burden of patients. However, its role might vary according to cultural context. Our study aimed to analyze religiosity, and its clinical, psychological and socio-demographic correlates in ALS patients and controls, comparing two European countries with different cultural backgrounds.

**Methods:**

268 Polish and German ALS patients, including 18 with locked-in syndrome (LIS) and 198 healthy controls (HC) were interviewed about religiousness, quality of life (Qol), depression, functional status and pain. A follow-up was conducted on 71 patients.

**Results:**

Polish subjects had a significantly higher level of public, private and general religiosity than the German sample. Importantly, we found no difference in total and public religiousness between ALS patients and HC within either population. Only the private religiousness was significantly higher in German patients compared to controls. In the same sample, private religiousness correlated with functional impairment due to disease progression. In ALS groups and LIS patients, religiousness did not correlate with any disease-associated factors: disease duration, pain, Qol or depression. Follow-up comparisons in the ALS group revealed worsening functional status, increased depression and no significant change in religiosity.

**Conclusions:**

Religiosity was linked to the cultural background rather than ALS. Generally, it did not correlate with clinical, psychological and socio-demographic parameters and was stable throughout disease progression. The only exception was the relationship between the functional decline and private religiosity among German patients.

**Supplementary Information:**

The online version contains supplementary material available at 10.1007/s11136-023-03383-4.

## Introduction

Amyotrophic lateral sclerosis is a fatal, neurodegenerative disease of both upper and lower motor neuron. Starting with a variable degree of weakness and wasting of the limb, facial or respiratory muscles, the disease inevitably leads to quadriplegia, anarthria, aphagia and respiratory failure. Although a number of factors predict survival, the disease prognosis remains poor in an individual patient [1]. Accordingly, about half of the patients die within 30 months from the first symptoms, and only ≤ 10% survive over 10 years [1, 2]. While during the course of the disease approx. 50% of patients show mild language and executive dysfunction, only 10–15% fulfill the criteria for frontotemporal dementia [3]. In the terminal stage, patients may develop a locked-in syndrome (LIS) characterized by loss of mobility and communication with preserved consciousness and sensation [4]. Currently, there is no efficient causative treatment for ALS. Two registered drugs, riluzole and edaravone, are only able to slightly prolong survival or reduce disease progression [5].

Considering the complexity of the physical problems, patients are in need of medical, physical, psychological and spiritual aid. Religion is perceived as an important source of psychological support [6]. It is associated with several aspects of mental and/or physical health, providing coping strategies, influencing well-being, depression, anxiety and health behaviors [7]. Religion was previously found to determine well-being in patients with ALS (PALS) [8–10], but the external and internal factors able to facilitate psychosocial adaptation, may vary between countries [11]. Given the fatal nature of ALS, we hypothesized that religiosity was a universal source of consolation and/or support in the affected individuals. Therefore, we were interested to see (1) if PALS were more religious than healthy individuals (HCs), (2) if living in different countries with their cultural and religious backgrounds could affect the overall religiosity of PALS, (3) whether particular socio-demographic factors, as well as clinical stage correlated with religiosity in PALS, (4) if religiosity changed along the disease progression, (5) whether individual’s religiosity was linked to depression and quality of life (QoL). To conduct the study, we chose Germany and Poland, two neighboring European countries with different socio-, demographic and religious backgrounds. While Germany after World War II was a part of Western Europe with increasing economic status, gradual laicization, liberalization and loosening family ties, in Poland under the communist regime similar processes were much slower. Additionally, a vast majority of the Polish population perceived family as a synonym for support and safety, while religion—a long-lasting source of strength in regaining individual and societal freedom. Also, Germany was originally protestant or catholic, while Poland after the World War II was much less diverse and Catholicism was a clearly dominant religion. These differences are still apparent today when Christians constitute approximately 50% of the German and 93% of the Polish society with 36% atheists in Germany and 5% in Poland [12]. In view of the above, it was important to analyze the patients’ religiousness on the background of healthy individuals from the same society. Hence, the overall aim of the study was to analyze religiosity and its links with sociodemographic, clinical and psychological factors in PALS and HC from the neighboring European countries with contrasting cultural backgrounds and patterns of religiosity.

## Patients and methods

### Patients

Two hundred and sixty-eight PALS were consecutively recruited at a single reference Polish (*n* = 133) and German (*n* = 135) university in-/outpatients clinics between 2014 and 2018. They constituted approximately 80% of all patients consulted at both centers fulfilling the inclusion criteria. The most frequent reasons for not participating were physical/mental fatigue, early state pre- or post-PEG insertion or qualification for NIV/IV (for hospitalized patients) or unwillingness to participate. The inclusion criteria were: age > 18 years, clinically definite, probable or probable laboratory-supported ALS according to the El Escorial revised criteria [13], time since diagnosis > 3 months, Polish/German mother tongue. The exclusion criteria were: dementia and other neurological/general conditions that could physically/psychologically influence the study outcome (e.g., stroke or bipolar disorder). The general group (GG) consisted of 115 Polish and 135 German patients; 38 Polish and 33 German patients were also available for a follow-up interview (15.53 ± 4.43 and 14.23 ± 5.52 months, respectively). There was an additional subgroup of Polish PALS with locked-in syndrome (LIS, *n* = 18), whose recruitment process was previously presented [14].

The healthy control group (HC) consisted of 198 age-, gender-, and education-matched individuals from the respective countries (*n* = 98. Poland and *n* = 100, Germany) without neurodegenerative and psychiatric diseases. Subjects were invited to the study via personal communication (ie. colleagues or hospital staff not involved in the study, accompanying persons of patients consulted with benign/transient conditions ie. headache, discopathy etc.) or social networks (facebook, What’s app).

All subjects signed informed consent prior to inclusion in the study. Patients in LIS provided informed consent using an eye-tracking system in the presence of a rater and another witness not related to the study. All interviews were conducted in patients’ native languages. The study was approved by the local Ethical Committees (Poland KB/138/2013; Germany 19/12) in accordance with the ethical standards of the 1964 Declaration of Helsinki and its amendments.

See Tables 1, 2 and 3 for demographic and clinical characteristics of ALS patients and controls.

**Table 1:** Between-group comparisons for demographic data, wellbeing and religiosity in Polish and German patients with amyotrophic lateral sclerosis and healthy controls

	N				Means				Standard deviations				Country x Group ANOVA^+^	LIS			
	Germany		Poland		Germany		Poland		Germany		Poland						
	ALS	HC	ALS	HC	ALS	HC	ALS	HC	ALS	HC	ALS	HC		N	M	SD	LIS vs ALS PL^++^
Age (years)	135	98	115	100	62.62	60.02	59.63	59.69	11.20	6.18	11.70	13.28	ns	18	58.89	9.49	818
Education (years)	135	97	106	100	13.17	12.70	13.76	13.77	2.97	2.73	3.66	3.10	PL > DE**	18	14.64	2.98	0.194
QoL (ACSA)	134	98	112	100	− 0.22	1.43	− 0.12	1.24	2.65	1.55	2.51	1.74	ALS < Control***	17	0.29	3.16	0.412
Pain (frequency)	124	98	98	100	3.29	2.80	3.38	3.73	2.17	1.53	2.30	1.74	PL > DE*; Interaction*	14	3.89	2.14	0.644
Pain (intensity)	123	98	98	100	2.22	2.30	2.13	2.59	1.35	1.11	1.25	0.79	ALS < Control*	14	3.21	1.05	0.002
Depression (ADI)	134	98	106	100	24.52	18.61	25.28	23.93	6.62	4.59	4.99	4.76	ALS > Control***, PL > DE***, Interaction***	18	25.61	4.72	0.935
public religiosity	135	98	115	100	4.76	4.48	5.62	5.78	2.24	2.03	1.88	2.09	PL > DE***	18	5.78	1.67	0.559
private religiosity	135	98	115	100	4.54	3.80	5.17	5.10	1.62	1.56	1.51	1.47	ALS > Control**, PL > DE***, Interaction*	18	4.67	0.49	0.406
total religiosity	135	98	115	100	9.30	8.28	10.79	10.88	3.48	3.34	3.11	3.34	PL > DE***	18	10.44	1.72	0.489

	*N* (total)				*N* (male)				% (male)				Country x Group Logistic regression	*N* (total)	*N* (male)	% (male)	
Gender	135	98	115	100	63	52	52	50	46.7	53.1	45.2	50.0	ns	18	5,00	27,80	0.205

*p < 0.05, **p < 0.01, ***p < 0.001

^+^Direction and significance of all effects in 2  × 2 between-subject ANOVAs with bootstrap *p*-values. Main effects described by group labels (ALS vs Control or Poland (PL) vs. Germany (DE). Interaction effects described in detail in the main text and in Part B of Supplementary Materials

^++^Comparisons using Mann–Whitney test

**Table 2:** Comparison of continuous clinical characteristics across ALS groups

	ALS Germany			ALS Poland			Germany vs Poland^+^	LIS			ALS PL vs LIS^++^
	*N*	*M*	SD	*N*	*M*	SD	*p*	*N*	*M*	SD	*p*
Disease duration (months)	135	23.21	11.39	115	26.32	13.92	0.057	18	144.39	218.62	< 0.001
Time since diagnosis (months)	133	11.06	7.11	115	11.12	8.52	0.958	18	73.50	43.69	< 0.001
ALSFRS-R	134	34.36	8.07	110	34.79	6.50	0.642	18	2.44	3.35	< 0.001

*ns* not significant, *ALS* amyotrophic lateral sclerosis, *LIS* polish ALS patients with locked-in syndrome, *ALSFRS-R* ALS Functional Rating Scale revised form

^+^Significance of bootstrap independent sample *t*-test

^++^Significance of Mann–Whitney test results

**Table 3:** Polish and German patients with amyotrophic lateral sclerosis—comparisons of dichotomous clinical characteristics

	ALS Germany			ALS Poland			Germany vs Poland^+^	LIS			ALS PL vs LIS^+^
	*N* (total)	*N*	%	*N* (total)	*N*	%	*p*	*N* (total)	*N*	%	*p*
PEG	135	16	11.9	111	6	5.4	0.115	18	18	100	< 0.001
NIV	135	49	36.3	111	6	5.4	< 0.001	18	2	11.1	0.309
IV	135	0	0	111	0	0	–	18	16	88.9	< 0.001

*ALS* amyotrophic lateral sclerosis, *LIS* Polish ALS patients with locked-in syndrome, *PEG* percutaneous endoscopic gastrostomy, *IV* invasive ventilation, *NIV* non-invasive ventilation

^+^Significance of Fisher’s exact test

The study was performed by neurologists specialized in neuromuscular diseases and clinical psychologists. Interviews were performed during in-patient or following outpatient visits. LIS patients were interviewed at home; they provided answers to interview questions using conventional eye-tracker systems or BCI systems as previously reported [14]. Patients’ data were anonymised according to the EU General Data Protection Regulation [15]; anonymisation lists were kept password-protected. The database used for further analysis did not contain sensitive data enabling subject identification.

### Clinical data

Functional/clinical status was assessed with the ALS Functional Rating Scale-revised (ALSFRS-R), ranging from 0 (locked-in state) to 48 (no functional impairment) [16]. The percentage of patients who were supported by percutaneous endoscopic gastrostomy (PEG), invasive (IV) and non-invasive ventilation (NIV) is presented in Table 3.

### Religiousness

Religiousness was quantitatively assessed with the Idler’s religiosity scale (IIR), a four-item instrument which measures public, private and general religious involvement. The public part concerns attendance at religious services and the number of community members known to the respondent. The private part represents a subjective religious experience. The score of public (2–10) and private religiousness (2–7), add up to the total score between 4 (the least religious) and 17 (the most religious) [17]. Cronbach alpha values for German and Polish samples were: 0.82 and 0.81 for total, 0.67 and 0.57 for public and 0.88 and 0.85 for private religiousness.

### Quality of life and depression measures

Global QoL was measured by the anamnestic comparative self-assessment (ACSA), which allows to estimate overall well-being using a -5 to 5 scale with the extreme scores labeled as the worst (− 5) and best (5) experience in the subject’s life. [18, 19]. Depression was measured with the ALS-Depression-Inventory 12 Items (ADI-12; numeric scores between 12 and 48) where higher results indicated lower mood [20]. The reliability of ADI-12 was satisfactory in both groups (Cronbach alpha = 0.77 and 0.89 in Polish and German participants, respectively).

Due to lack of validated translations, for the purpose of the study ACSA and ADI-12 were adapted from original English versions. Translation accuracy and content validity were ensured by carrying out a procedure consisting of translation, independent back-translation, and final verification performed by experienced clinical psychologists fluent in English and the target language (See Supplementary Materials Part A).

As a single-item measure, ACSA does not allow for the assessment of internal consistency. It is, however commonly used in severely ill subjects and shows satisfactory psychometric properties (see: [18] for detailed analysis). Both translated tools were previously used by our group [11]. The same ACSA adaptation was used in the phase 3-clinical study ORARIALS-01/02 in PALS and caregivers [21].

### Pain

Pain level was expressed on a quantitative scale (frequency: How often do you feel pain? 1 = never to 6 = every day; intensity: How severe is this pain? 1 = lack of pain to 6 = unbearable pain).

### Statistical analysis

To estimate the effects of Country (Poland vs Germany) and Diagnosis (PALS vs healthy controls) on continuous outcomes 2-way ANOVA was used. Logistic regression in the same design tested hypotheses concerning the distributions of gender across groups. In two-group or two-measurement comparisons, *t*-tests were performed with the exception of analyses involving the LIS group which, due to the limited sample size, were based on the Mann–Whitney test. The independence of nominal variables was tested using Fisher exact test. All correlations were computed using Spearman’s rho coefficient. Due to the non-normality of many of the outcome measures and the presence of outliers, the significance of all parametric tests was based on bootstrap *p*-values (2000 samples). For statistical inference, *p*-values < 0.05 were considered significant.

This study constitutes a part of a scientific project „Needs in ALS” which focuses on the end-of-life dilemmas of PALS. Hence, initial power calculations were related to the primary goals of the project and were described in other publications [11].

## Results

### Group characteristics

Patients from the general group (GG, *n* = 115 Polish and *n* = 135 German patients) shared a comparable age, gender, education and functional status. Among LIS patients, there was a higher proportion of males, a significantly longer disease duration and a lower functional state as compared to the GG. There was also a higher frequency of NIV among German patients.

### Religiosity

The main effect of Illness on religiosity (ALS versus controls) was observed for private but not for public religiosity, with higher scores in PALS compared to controls. The difference was however modified by an interaction effect with the Country, namely the level of private religiousness did not significantly differ between patients and controls in the Polish sample, whereas it was significantly higher in German PALS compared to the German HC. No other main or interaction effects involving Diagnosis were present.

**Fig. 1:**
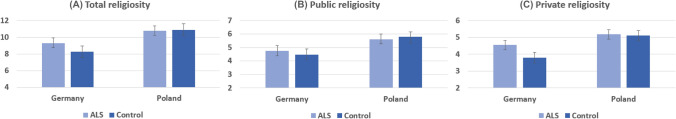
Comparison of mean religiousness in Polish and German patients with amyotrophic lateral sclerosis and healthy controls. Error bars depict 95% bootstrap confidence intervals

There was a significant effect of the Country (Poland versus Germany) with higher scores of public, private, and general religiosity in Polish compared to German patients (Table 1, Fig. 1).

### Socio-demographic correlates of religiosity across groups (Table 4)

**Table 4:** Spearman's rho coefficients and sample sizes describing correlations between the demographic, psychological, and clinical variables and three indices of religiosity in all tested groups

	Germany								Poland								LIS—Poland			
	ALS				Control				ALS				Control							
	*N*	Public	Private	Total	*N*	Public	Private	Total	*N*	Public	Private	Total	*N*	Public	Private	Total	*N*	Public	Private	Total
Gender (f = 0, m = 1)	135	0.022	0.221*	0.127	98	0.188	0.256*	0.248*	115	0.190*	0.279**	0.260**	100	0.185	0.256*	0.215*	18	0.401	0.381	0.433
Age (years)	135	0.139	− 0.006	0.094	98	0.213*	0.128	0.193	115	0.162	0.274**	0.238*	100	0.310**	0.377***	0.355***	18	0.420	0.496*	0.499*
Education (years)	135	− 0.160	− 0.104	− 0.161	97	0.134	0.149	0.149	106	− 0.395***	− 0.377***	− 0.431***	100	− 0.131	− 0.178	− 0.170	18	− 0.373	− 0.268	− 0.354
Pain (frequency)	124	0.030	− 0.020	0.005	98	0.194	0.159	0.201*	98	− 0.063	− 0.143	− 0.077	100	0.141	− 0.055	0.056	18	− 0.076	− 0.213	− 0.152
Pain (intensity)	123	0.071	0.057	0.069	98	0.123	0.023	0.082	98	− 0.016	− 0.095	− 0.025	100	− 0.051	− 0.122	− 0.087	14	− 0.289	− 0.149	− 0.234
QoL (ACSA)	134	0.093	0.024	0.060	98	− 0.064	− 0.044	− 0.073	112	0.068	− 0.043	0.032	100	0.000	0.020	0.005	17	− 0.274	− 0.213	− 0.214
Depression (ADI)	134	− 0.104	0.041	− 0.023	98	0.177	0.090	0.143	106	− 0.004	0.069	0.036	100	− 0.11	− 0.12	− 0.119	18	0.493*	0.477*	0.463
Disease duration (months)	135	0.096	0.071	0.101					115	0.051	0.028	0.046					18	− 0.123	0.092	− 0.058
Time since diagnosis (months)	133	0.105	0.004	0.069					115	− 0.023	0.007	− 0.006					18	0.027	0.162	0.046
ALSFRS-R	134	− 0.077	− 0.210*	− 0.163					110	− 0.101	− 0.148	− 0.133					18	− 0.145	− 0.106	− 0.125

*ALS* amyotrophic lateral sclerosis, *LIS* Polish ALS patients with the locked-in syndrome, *ACSA* Global QoL measured using the anamnestic comparative self-assessment. *ADI* depression score measured by ALS-Depression-Inventory 12 Items. *Total* public and private religiosity based on Idler’s religiosity scale (IIR). *ALSFRS-R* functional status measure—ALS Functional Rating Scale—Revised form

*p < 0.05, **p < 0.01, ***p < 0.001

#### ALS patients (GG)

There was a positive correlation between age and private or total religiosity among Polish but not German PALS. A negative correlation between religiosity and years of education for public, private and overall religiosity was found in the Polish but not in the German population.

#### Healthy controls (HC)

There was a positive correlation between age and public religiosity in German HC and between age and public, private and overall religiosity in the Polish HC. There was no correlation between years of education and religiosity in either Polish or German healthy subjects.

#### Locked in syndrome group (LIS)

There was a positive correlation between private and total religiosity and age but not with years of education.

### Clinical correlates of religiosity across groups (Table 4)

#### ALS patients (GG)

In neither Polish nor German PALS was there a correlation between religiousness (public/private/overall) and disease duration, global QoL (ACSA), depression (ADI-12) or pain. Functional state (ALSFRS-R) negatively correlated with private religiousness in the German patients.

#### Healthy controls (HC)

There was no correlation between religiousness (public/private/overall), global QoL, depression and pain in either Polish or German HC (except for a weak positive correlation between total religiousness and frequency of pain in the German HC).

#### Locked in syndrome (LIS)

There was a positive correlation between public and private religiosity and depression. There was no correlation between religiousness, disease duration, ALSFRS-R, global QoL, pain and education**.**

### Follow-up data

At the follow-up visit, PALS presented with a functional decline (difference of means: − 12 among the Polish and − 9 in the German patients) and increased depression (difference of mean: 3.6 and 2.6, respectively) as compared to the initial data. There was no significant difference between religiosity, global QoL and pain with disease duration. The results did not differ between Polish and German patients (Table 5).

**Table 5:** Descriptive statistics and comparisons of religiosity, functional state, pain and depression scores in the follow-up analysis of Polish and German ALS patients

	Poland						Germany					
	*N*	1st measurement		2nd measurement		*p*^+^	*N*	1st measurement		2nd measurement		*p*^+^
		*M*	SD	*M*	SD			*M*	SD	*M*	SD	
ALSFRS-R	35	37.66	6.12	25.77	9.96	< 0.001	33	37.18	5.31	28.18	8.42	0.001
Pain (frequency)	29	3.1	2.29	3.24	2.39	0.717	32	3.41	2.11	4.00	2.03	0.193
Pain (intensity)	29	2.24	1.48	2.14	1.38	0.725	32	2.16	1.32	2.50	1.30	0.227
QoL (ACSA)	38	− 0.39	2.35	− 1.05	2.48	0.309	33	− 0.03	2.65	− 0.45	2.48	0.278
Depression (ADI)	28	24.57	4.69	28.14	4.08	< 0.001	33	23.24	5.90	25.85	7.58	0.012
Public religiosity	37	5.14	1.69	5.24	1.92	0.661	33	4.94	1.27	4.88	1.49	0.779
Private religiosity	38	4.74	1.45	4.61	1.79	0.515	33	4.73	0.57	4.58	0.56	0.197
Total religiosity	38	9.95	2.92	9.71	3.65	0.602	33	9.67	1.31	9.45	1.66	0.446

*ALS* amyotrophic lateral sclerosis; The functional status measure: ALS Functional Rating Scale revised form (ALSFRS-R). Global QoL measured using the anamnestic comparative self-assessment (ACSA). Depression measure: ALS-Depression-Inventory 12 Items (ADI-12). Total, public and private religiosity measured Idler’s religiosity scale (IIR)

^+^Significance of bootstrap dependent sample *t*-test

## Discussion

ALS is a strongly handicapping disease that leads to a loss of physical autonomy in a relatively short time. The patients are confronted with a progressive functional decline with full awareness, as cognitive impairment is rare. Physical disability has been shown to prevent active participation in religious gatherings [22]. We therefore focused on both private and public religiousness based on patients’ self-assessment of the depth of their religiousness, strength and comfort obtained from religion, as well as attendance at religious services and acquaintance with other congregation members.

As a coping resource, religiousness has been shown to correlate with life satisfaction in health and disease across the globe [23, 24]. Since it constitutes an important element of the cultural background of a society [25], the impact of religiousness on several aspects of life may also significantly vary between countries [26].

### Are patients with ALS more religious than healthy individuals?

Generally, we did not find PALS to be more religious than HC. The effects of ALS diagnosis were apparent only in the German population, where the sick individuals reported a significantly higher private religiousness (which correlated with functional impairment) compared to HC. In Polish patients, an analogous difference between the sick and the healthy was not significant. For the remaining indices, no such relationship was found.

The specific effect concerning private religiosity of the German PALS could reflect genuine changes to the spiritual life of affected patients [26, 27]. It might be the case that similar effects in Poland are more difficult to observe, masked by the overall—much higher—societal levels of religiousness. Indeed, approximately 60% of Polish adults report attending religious services ≥ 1amonth, 25% pray daily and approximately 40% find themselves “highly religious” as compared to 24%, 9% and 12% in Germany, respectively [28]. At the same time, that above reported effect in German PALS remains isolated, as no other results in our study support deepening of religiousness as a correlate of ALS diagnosis and progression.

### May living in different countries (with their diverse cultural and religious backgrounds) influence the overall religiosity of ALS patients?

We found significantly higher indices of public, private, and general religiosity in the Polish compared to the German group. The results of PALS closely mimic the patterns observed in the general population, which is also coherent with previous findings on the public religiosity in a smaller group of Polish and German PALS (*n* = 83 in each group) [11].

### Do socio-demographic and clinical factors correlate with religiosity in patients with ALS?

Among Polish, but not German PALS, religiousness was significantly linked to age and education. It also significantly correlated with age in both control groups suggesting the influence of socio-cultural factors and/or cohort effects. The association between religion and age has previously been shown in American (*n* = 122) and British (*n* = 173) reports, which additionally revealed that private religious activity was associated with age in elderly patients with chronic diseases [29, 30].

As for the level of education, similarly to our results, a large American study (*n* = 2537) showed a negative correlation between private religiosity and education, but only a marginal positive association with public religiosity [31]. In a meta-analysis of 83 publications, Zuckerman et al. found a negative correlation between religiosity and intelligence, not directly modified by the level of education, but dependent on a rational and analytical cognitive style [32].

Interestingly, in the present study we observed no relationship between public religiousness and functional impairment in either Polish or German PALS. The findings differ from earlier results obtained in the elderly, where the attendance in religious gatherings (public religiousness) was associated with a lower level of functional disability [17, 22, 33]. There was an important impact of physical disability on declining attendance in religious practices (disability as a negative short-term factor on attendance) [22]. Our results can be explained by a younger age of the interviewed individuals (mean 59.6 in the Polish and 62.6 in German PALS) not defined as an elderly population and easier access to online/remote ways of worship as compared to studies from the twentieth century. The above social changes could not however explain the fact that in our general patients’ population, we found no association between private religiousness and functional impairment either. A negative correlation between private religiousness and ALSFRS-R was only observed in the German subgroup. Again, it can either be explained by an increased disease-related spirituality in patients from more secular populations [26] or a higher, culture-based importance of physical health among the Germans [34] as compared to the Poles [35]. A study comprising individuals from several countries, or analyzing German attitudes towards functional impairment in cases of other diseases would be needed to confirm the above hypotheses.

In summary, the correlations between demographic characteristics and religiousness, do not suggest any ALS-related specificity, as they are quite similar in comparison with HC. The differences between Germany and Poland in the role of age might probably be explained by the cohort effects (which, in a cross-sectional study are confounded) and differences in the trajectories of liberalization and modernization of both societies [36].

### Does religiosity change along the disease progression?

We found no correlation between religiousness and disease duration in either Polish or German PALS. Importantly, there was also no increase in religiosity in the follow-up analysis, suggesting that religiousness and patterns of religious practices might be fairly stable throughout the disease course. While one can argue that the disease duration among the interviewed PALS (mean 26 months in Poland and 23 months in Germany) might be too short to assess the effects of the adaptation process, the results from the follow-up data (interview after a mean of 15.5 months in Poland and 14 months in Germany) and a small group of Polish patients with LIS (mean disease duration 73.5 months) support the conclusion about lack of shifts in religiousness over time. Our results go in line with previous analyses by Walsh et al. and Bremer et al., who showed no significant change in religiousness among PALS over time/disease progression [9, 37]. Robbins et al. found only a subtle change in religiosity and QoL despite a significant decline in physical function among PALS [38]. Similar results were found in Parkinson’s disease, where the disease progression did not lead to changes in religiosity [39].

Although frequently neglected, pain occurs in a considerably high number of PALS at every disease stage [40]. In the current study, we found no correlation between religiousness and pain incidence or intensity among PALS and controls from both countries (except for a weak positive correlation between total religiousness and frequency of pain in the German HCs). Similar results were obtained in North American and Brazilian patients with musculoskeletal [29] or pelvic pain, respectively [41].

### Is religiosity linked to depression and the quality of life?

Several studies discussed the buffering effects of religiousness on depression [42, 43]. We didn’t show correlations between religiousness (public/private/overall) and depression in PALS and HC either in Poland or Germany. According to the systematic review on depression and religiosity/spirituality covering 444 studies performed within nearly 50 years, there was an inverse association between depression and religiousness, namely the less depressive individuals were more religious [44]. Koenig et al. showed that religious involvement was linked to reduced time to remission in the depressed individuals with heart failure and/or chronic pulmonary disease [45]. In PALS with LIS, contrary to the above-cited studies, we even found a positive correlation between religiousness and depression. Beside the obvious uncertainty of the estimations based on a small sample, several mechanisms might be responsible for this phenomenon. The more religious individuals with ALS could be more vulnerable to depression, as religion might increase guilt by focusing on sin [7]. Conversely, depressed individuals with LIS/ALS may develop greater spiritual needs and/or search for consolation in religion. Simmons et al. emphasized the role of psychological and existential factors in patient’s QoL: they found a positive correlation between religiosity and QoL [46] in a group of Pensilvanian PALS (*n* = 96, mean age of 57.8 years) with a slightly longer disease duration (31.8 months as compared to 26 and 23 months in the current study in Poland and Germany, respectively).

Also contrary to the above-mentioned and other studies in the elderly [47], we found no correlations between global QoL and religiousness either in the GG of Polish and German patients, or in patients with LIS. There was also no such correlation in the HCs from either country. The patients in our study did not show any changes in QoL along the disease progression, consistently with other studies showing that QoL was maintained despite a deterioration of physical function [4, 37, 38].

Even though religiousness does not seem directly related to QoL indices in PALS and controls from Poland and Germany, a number of studies emphasize the importance of religious needs for many PALS [10], as well as a supportive role of faith-based intervention in individuals for whom religiosity constitutes a significant part of life [37]. Still, it should be emphasized that the complex interplay of cohort effects and socio-cultural factors influencing the patterns of religious practices makes it challenging to generalize results across countries.

## Limitations

Most important limitations of our study stem from the declarative nature of the psychological measures, as well as problems inherent to cross-sectional studies comparing populations of different countries. Certainly, our analysis could benefit from including a broader range of variables capturing the socioeconomic background, objectively available and perceived social and practical support in dealing with the illness etc. Regrettably, such data was not gathered within this project. The study would also profit from a higher number of patients who completed the follow-up questionnaire and a longer observation time. However, that possibility is significantly limited due to the relentless disease progression and its fatal outcome. This last remark directs our attention toward more general methodological challenges, the most central being the problem of measurement invariance and psychometric properties of questionnaires. While the reliability and convergence validity of the tools could be assessed as satisfactory in our study, we had no chance to establish their invariance (both, with respect to the ALS vs HCs comparisons, as—even more so—between the language versions). That problem, to a large extent, determined the choice of the statistical methods, which focused on comparisons and correlations, without the use of more complex models allowing for better control of confounding, but resting on the assumptions that we considered untenable.

It is also worth noting that—given a low number of studies on religiosity in ALS—the empirical context used in the interpretations of the obtained results may not be fully adequate. Especially that the observations reported for other diseases or disabilities might not be exactly comparable to the devastating nature of ALS. In addition, in rapidly changing circumstances (geopolitical processes, COVID pandemic), it is particularly difficult to postulate the stability of psychological mechanisms strongly embedded in the complexity of social and cultural processes.

## Conclusions

Despite a severely handicapping and fatal character of ALS, our results—obtained in a large sample, using varied measures of religiosity and a broad range of its potential correlates—suggest no overall correlations between the clinical and psychological characteristics of ALS and religiosity. We also found hardly any systematic differences between the ALS and HCs when taking into account the subjects’ country of provenance. At the same time, religiousness was significantly linked to the country of origin, age and levels of education, which should be seriously taken into consideration in any comparative studies in PALS from varied cultural and social backgrounds. The only country-specific effect was the observation that private religiosity was higher in German PALS as compared to HCs, and that it correlated with functional impairment. That points out a potential role of religion in the process of coping with the disease in certain subgroups of patients (i.e. in more secular countries). That relationship certainly requires further validation concerning the broad range of limitations mentioned above. It is also important to note that our analysis treated patient groups as homogenous, so we cannot exclude that the spiritual support might be highly beneficial for at least some of them. Hence, to address the variety of individual needs when confronting a progressive life-shortening disease, the thorough care of PALS should address their religious needs respecting the cultural contexts [2, 11].

## Supplementary Information

Below is the link to the electronic supplementary material.

Supplementary file1 (PDF 47 KB)

Supplementary file2 (PDF 91 KB)
