# Supplementary material for: Religiosity in patients with amyotrophic lateral sclerosis, a cross-country comparison
Source: Qual Life Res. 2023 Mar 16;32(8):2235–46. doi: 10.1007/s11136-023-03383-4 (PMC10328897; doi:10.1007/s11136-023-03383-4)
Supplement: Supplementary file 1 — Supplementary file1 (PDF 47 KB) [file 11136_2023_3383_MOESM1_ESM.pdf]

Religiosity in patients with amyotrophic lateral sclerosis, a cross-country comparison.

Ciećwierska et al., Quality of Life Research, 2023

Supplementary Materials Part A.

Description of the Anamnestic comparative self-assessment (ACSA) and ALS-Depression-Inventory 12 Items (ADI-12) translation process

Due to the lack of validated translations, both scales were adapted from original English versions, and the process included: translation, independent back-translation and final verification.

The original English versions of the ACSA and ADI-12 were initially translated to Polish by team members trained in neurology and psychology, fluent in English and the target language. The translations were prepared following established guidelines [1].

Then, the forward translation was reviewed by an independent bilingual translator familiar with the phraseology and idiomatics of the Polish language. The clinical team members reviewed the suggested changes. Finally, the back-translation from Polish to English was made and compared to the original version to ensure translation quality.

References:

1. Sousa VD, Rojjanasrirat W. Translation, adaptation and validation of instruments or scales for use in cross-cultural health care research: a clear and user-friendly guideline. J Eval Clin Pract. 2011 Apr;17(2):268-74. doi: 10.1111/j.1365-2753.2010.01434.x. Epub 2010 Sep 28. PMID: 20874835
