# Supplementary material for: Religiosity in patients with amyotrophic lateral sclerosis, a cross-country comparison
Source: Qual Life Res. 2023 Mar 16;32(8):2235–46. doi: 10.1007/s11136-023-03383-4 (PMC10328897; doi:10.1007/s11136-023-03383-4)
Supplement: Supplementary file 2 — Supplementary file2 (PDF 91 KB) [file 11136_2023_3383_MOESM2_ESM.pdf]

Religiosity in patients with amyotrophic lateral sclerosis, a cross-country comparison.

Ciećwierska et al., Quality of Life Research, 2023

Supplementary Materials Part B.

Detailed statistics for 2x2 ANOVAs reported in Table 1.

For each of the dependent variables ANOVA/GLM results are presented, including the significance of Country, Diagnosis, and interaction effects. For significant interactions, the results of post-hoc test performed within each country group are reported.

Age

	SS	*df*	*MS*	*F*	*p*
Country	303.194	1	303.194	2.513	0.114
Diagnosis	178.619	1	178.619	1.48	0.224
Country * Diagnosis	194.447	1	194.447	1.612	0.205
Error	53571.74	444	120.657		

Education

	SS	*df*	*MS*	*F*	*p*
Country	74.882	1	74.882	7.646	0.006
Diagnosis	5.912	1	5.912	0.604	0.438
Country * Diagnosis	6.211	1	6.211	0.634	0.426
Error	4250.674	434	9.794		

ACSA

	SS	*df*	*MS*	*F*	*p*
Country	0.258	1	0.258	0.052	0.819
Diagnosis	247.573	1	247.573	50.357	0.000
Country * Diagnosis	2.142	1	2.142	0.436	0.510
Error	2163.214	440	4.916		

Pain frequency

	SS	*df*	*MS*	*F*	*p*
Country	27.112	1	27.112	6.978	0.009
Diagnosis	0.524	1	0.524	0.135	0.714
Country * Diagnosis	18.641	1	18.641	4.798	0.029
Error	1616.207	416	3.885		

Post-hoc comparisons				Bootstrap		
Country	Group 1	Group 2	Mean Difference	Bias	SE	p
Germany	ALS	CON	0.494	0.004	0.253	0.054
Poland	ALS	CON	-0.352	0.008	0.289	0.237

Pain intensity

	SS	*df*	*MS*	*F*	*p*
Country	1.114	1	1.114	0.83	0.363
Diagnosis	7.392	1	7.392	5.508	0.019
Country * Diagnosis	3.766	1	3.766	2.806	0.095
Error	556.957	415	1.342		

Depression (ADI)

	SS	*df*	*MS*	*F*	*p*
Country	995.85	1	995.85	33.992	0.000
Diagnosis	1421.9	1	1421.9	48.535	0.000
Country * Diagnosis	559.757	1	559.757	19.107	0.000
Error	12714.718	434	29.297		

Post-hoc comparisons				Bootstrap		
Country	Group 1	Group 2	Mean Difference	Bias	SE	p
Germany	ALS	CON	5.91	-0.039	0.732	0.001
Poland	ALS	CON	1.353	-0.017	0.685	0.050

Public religiosity

	SS	*df*	*MS*	*F*	*p*
Country	127.89	1	127.89	29.836	0.000
Diagnosis	0.402	1	0.402	0.094	0.760
Country * Diagnosis	5.478	1	5.478	1.278	0.259
Error	1903.199	444	4.286		

Private religiosity

	SS	*df*	*MS*	*F*	*p*
Country	103.367	1	103.367	43.339	0.000
Diagnosis	18.463	1	18.463	7.741	0.006
Country * Diagnosis	12.398	1	12.398	5.198	0.023
Error	1058.966	444	2.385		

Post-hoc comparisons				Bootstrap		
Country	Group 1	Group 2	Mean Difference	Bias	SE	p
Germany	ALS	CON	0.745	0.003	0.207	0.002
Poland	ALS	CON	0.074	0.004	0.195	0.694

Total religiosity

	SS	*df*	*MS*	*F*	*p*
Country	461.21	1	461.21	41.675	0.000
Diagnosis	24.311	1	24.311	2.197	0.139
Country * Diagnosis	34.358	1	34.358	3.105	0.079
Error	4913.661	444	11.067		

Gender

GLM - omnibus Test against the null model.

Likelihood Ratio Chi-Square	*df*	*p*
1.584	3	0.663

Tests of Model Effects	*Wald Chi-Square*	*df*	*p*
Country	0.225	1	0.636
Diagnosis	1.376	1	0.241
Country * Diagnosis	0.028	1	0.866
